# Development of Tumor Microenvironment-Responsive Nanoparticles with Enhanced Tissue Penetration

**DOI:** 10.3390/nano15221695

**Published:** 2025-11-09

**Authors:** Karin Kitamura, Ryo Matsui, Nagisa Itagaki, Yuka Takeuchi, Hana Fukuda, Ken-Ichiro Tanaka, Susumu Hama

**Affiliations:** 1 Laboratory of Pharmaceutical Technology, Faculty of Pharmacy, Musashino University, Tokyo 202-8585, Japan; 2 Department of Biophysical Chemistry, Kyoto Pharmaceutical University, Kyoto 607-8414, Japan; 3 Laboratory of Bio-Analytical Chemistry, Faculty of Pharmacy, Musashino University, Tokyo 202-8585, Japan; 4 Research Institute of Pharmaceutical Sciences, Musashino University, Tokyo 202-8585, Japan

**Keywords:** tumor microenvironment, tissue penetration, liposomes

## Abstract

Liposomes modified with slightly acidic pH-sensitive peptides (SAPSp-lipo) are effectively delivered to tumor tissues, followed by cellular uptake in the tumor microenvironment. Although SAPSp-lipo can penetrate tumor tissues via the interspace route between cancer cells and the extracellular matrix (ECM), penetration needs to be enhanced to deliver liposomes into tumor cores comprising malignant cancer cells. To enhance the intratumoral penetration of SAPSp-lipo, we focused on the internalizing RGD peptide (iRGD), which can penetrate tumor tissue, differing from the penetration mechanism of SAPSp. In this study, we developed liposomes modified with iRGD-conjugated SAPSp (SAPSp-iRGD-lipo). Compared with SAPSp-lipo, SAPSp-iRGD-lipo was delivered to deeper regions within both spheroids and tumor tissues. The enhanced penetration was suppressed by a co-treatment with a Neuropilin-1 inhibitor, and the fluorescence signals from intratumorally injected SAPSp-iRGD-lipo were localized in Neuropilin-1-expressing regions, indicating a Neuropilin-1-mediated tumor penetration. Moreover, SAPSp-iRGD-lipo reduced F-actin formation in monolayered cells and was not localized in F-actin-rich regions in tumors, suggesting that SAPSp-iRGD-lipo facilitates tumor penetration through actin depolymerization. In addition, anticancer siRNA delivered by SAPSp-iRGD-lipid nanoparticles effectively induced apoptosis in cells under slightly acidic conditions. Taken together, SAPSp-iRGD-modified nanoparticles represent a novel class of tumor-penetrable and microenvironment-responsive drug carriers capable of efficient intratumoral delivery and therapeutic activity.

## 1. Introduction

To achieve effective chemotherapy for solid tumors, it is imperative to develop drug carriers that can modulate systemic pharmacokinetics and intratumoral and intracellular drug dynamics [1]. Currently, polyethylene glycol (PEG)-modified carriers, which exhibit extended circulation times, are extensively employed to passively deliver drugs to tumors through the enhanced permeability and retention (EPR) effect [2]. However, PEGylated carriers have limitations, including a low cellular uptake efficiency [3] and the Accelerated Blood Clearance (ABC) phenomenon upon repeated administration [4]. This necessitates the development of alternative PEG-free delivery systems. In contrast, cationic carriers, which demonstrate a high cellular uptake efficiency, tend to interact with biological components in the bloodstream, leading to reduced circulation times and suboptimal tumor accumulation [5].

Liposomal formulations have been extensively investigated as versatile nanocarriers for improving drug delivery efficiency. Previous comprehensive reviews have summarized their composition, preparation methods, and physicochemical properties that influence therapeutic performance [6]. More recently, advances in liposome-based nanocarriers for targeted and controlled delivery were highlighted by Alavi et al. [7], underscoring the continuing evolution of liposome technologies. These studies provide the foundation for the development of pH- and peptide-responsive liposomal systems explored in the present work.

In our previous study, we developed an innovative liposomal carrier modified with a slightly acidic pH-sensitive peptide (SAPSp) [8,9,10]. This carrier is engineered to respond to a slightly acidic tumor microenvironment by altering its surface charge from negative to positive. At physiological pH, SAPSp-modified liposomes (SAPSp-lipo), which possess a negative surface charge, accumulate in tumors, similar to PEGylated liposomes (PEG-lipo) [8]. When exposed to the slightly acidic conditions present within tumors, the surface charge becomes positive, thereby facilitating an enhanced cellular uptake and the efficient cytoplasmic delivery of encapsulated drugs [8].

Furthermore, SAPSp-lipo infiltrates tumors through the cell–extracellular matrix (ECM) pathway by inducing actin depolymerization in cancer cells [10]. Nonetheless, its intratumoral penetration remains inadequate for the effective delivery to deeply situated cancer cells, suggesting the need for further enhancements. Recent studies have introduced various strategies to enhance tumor penetration through the physicochemical and biological modulation of nanocarriers, including charge conversion and the use of tissue-penetrating peptides. In particular, the conjugation of iRGD peptides or related CendR motifs can significantly enhance tumor penetration and drug delivery efficiency [11,12,13]. Incorporating these concepts, the present work aims to design SAPSp-iRGD-lipo as a dual-function carrier that integrates pH responsiveness and iRGD-mediated tissue penetration. Upon proteolytic cleavage within the tumor microenvironment, iRGD reveals its CendR motif, which subsequently binds to and activates neuropilin-1 (NRP-1), initiating trans-tissue transport [14,15,16]. The present study introduces the novel concept of a dual-functional peptide-modified liposome (SAPSp-iRGD-lipo) that integrates the pH-responsive behavior of SAPSp with the NRP-1-mediated tumor-penetrating ability of iRGD. This design enables a dynamic adaptation to the tumor microenvironment, allowing for both efficient blood circulation and deep tumor penetration—overcoming the inherent trade-off between stability and permeability observed in conventional liposomal systems.

In this study, we developed SAPSp-iRGD-modified liposomes (SAPSp-iRGD-lipo) and investigated their tissue-penetrating capabilities and pH-responsive characteristics. Furthermore, we encapsulated antitumor siRNAs within SAPSp-iRGD-modified lipid nanoparticles (SAPSp-iRGD-LNPs) and evaluated their cytotoxic efficacy against cancer cells.

## 2. Materials and Methods

### 2.1. Materials and Animals

The mouse melanoma cell line B16−F1 and human melanoma cell line A375 were obtained from DS Pharma Biomedical Co., Ltd. (Osaka, Japan). Human colorectal adenocarcinoma Caco-2 cells were obtained from the RIKEN BioResource Research Center (Ibaraki, Japan). Egg phosphatidylcholine (EPC; Product No. available upon request NOF Corporation) was purchased from the NOF Corporation (Tokyo, Japan). 1,2-Dioleoyl-3-trimethylammonium propane (DOTAP; Product No. 890890P), 1,2-dioleoyl-sn-glycero-3-phosphoethanolamine (DOPE; Product No. 850725P), and 1,2-dioleoyl-sn-glycero-3-phosphoethanolamine-N-(lissamine rhodamine B sulfonyl) (Rh-PE; Product No. 810150P) were obtained from Avanti Polar Lipids (Alabaster, AL, USA). Matrigel^®^ Matrix Basement Membrane Phenol-Red Free was purchased from Corning Life Sciences (Tewksbury, MA, USA; Product No. 35623). Fluorescent dyes, including 3,3′-Dioctadecyl-5,5′-Di (4-Sulfophenyl) Oxacarbocyanine, Sodium Salt (DiO; product No. D7778), LysoTracker Green DND-26 (product No. L7526), Alexa Fluor^®^ 594 F(ab’)_2_ fragment of rabbit anti-goat IgG (H+L) (product No. A11080), and Alexa Fluor^®^ 488 goat anti-rabbit IgG, were obtained from Invitrogen (product No.A11008) (Carlsbad, CA, USA). Goat anti-Neuropilin-1 antibody was purchased from R&D Systems (Minneapolis, MN, USA; product No. AF556), and rabbit anti-ZO-1 IgG was obtained from Santa Cruz Biotechnology (Dallas, TX, USA; product No. sc-10804). Rhodamine phalloidin was purchased from Cytoskeleton, Inc. (Denver, CO, USA; product No. PHDR1). DiR (1,1′-dioctadecyl-3,3,3′,3′-tetramethylindotricarbocyanine iodide) was purchased from PerkinElmer (XenoLight DiR, product No. 125964; Revvity Inc., Boston, MA, USA). Stearylated peptides, including SAPSp (stearyl-GGGGHGAH-EHAGHEHAAGEHHAHE-NH_2_), iRGD (stearyl-GGGG(C)RGDKGD(C), circularized at cysteine), and SAPSp-iRGD (stearyl-GGGGHGAH-EHAGHEHAAGEHHAHEGGGG(C)RGDKGD(C), circularized at cysteine), were synthesized by Scrum, Inc. (Tokyo, Japan). Anti-luciferase siRNA (5′-GCGCUGCUGGUGCCAACCCTT-3′, 5′-GGGUUGGCACCAGCAGCGCTT-3′) and anti-human KIF11 siRNA (5′-CUGAAGACCUGAAGACAAUTT-3′, 5′-AUUGUCUUCAGGUCUUCAGTT-3′) were synthesized by Invitrogen. Five-week-old male Hos:HR-1 hairless mice and BALB/cSlc-nu/nu mice were purchased from Sankyo Labo Service Corporation, Inc. (Tokyo, Japan). All animal procedures were conducted in accordance with the institutional guidelines for the care and use of laboratory animals and were approved by the Institutional Animal Care and Use Committee (IACUC) of Musashino university (Approval No. [08-A-2024 and 08-A-2025]). Anesthesia was performed using sodium pentobarbital, and all efforts were made to minimize the suffering of the animals.

### 2.2. Preparation of Liposomes and Lipid Nanoparticles

Liposomes were prepared using a simple hydration technique, as described previously [10,17]. EPC and DOTAP, dissolved in ethanol, were combined at a molar ratio of 7.6:1 and subsequently dried under nitrogen gas to form lipid films. The films were hydrated with PBS(–) (final lipid concentration: 10 mM) and subjected to sonication using a bath-type sonicator. Surface modification was achieved by incubating the liposomes with 5 mol% stearylated SAPSp, iRGD, or SAPSp-iRGD for 30 min, resulting in SAPSp-lipo, iRGD-lipo, and SAPSp-iRGD-lipo, respectively. Lipid nanoparticles (LNPs) were prepared using the butanol dilution method, as described previously [18]. EPC, DOTAP, and DOPE were mixed at a molar ratio of 5.6:1:2. siRNA was diluted in RNase-free water, added under vortexing, and rapidly diluted in 2 mL citrate buffer. The resulting suspension was filtered using Amicon^®^ Ultra-15 Centrifugal Filter Units (Ultracel^®^-100K, Merck Millipore Ltd., Burlington, MA, USA) with 7 mL PBS(–) and centrifuged at 1000× *g* for 15 min. The flow-through was discarded, and the retentate was washed with 10 mL PBS(–) through a second centrifugation (25 min, 1000× *g*). SAPSp-iRGD modification was accomplished by a 30 min incubation with 5 mol% stearylated SAPSp-iRGD. The encapsulation efficiency of siRNA was quantified using the RiboGreen^®^ Assay (Thermo Fisher Scientific Inc., Waltham, MA, USA). The particle size and zeta potential were measured using a Zetasizer Nano (Malvern Instruments Ltd., Malvern, Worcestershire, UK).

### 2.3. Spheroid Penetration of Liposomes

A375 cells were cultured in DMEM supplemented with 10% FBS and 1% Matrigel^®^ in 96-well NanoCulture Plates (SCIVAX Life Sciences, Kanagawa, Japan) for 96 h under humidified 5% CO_2_ at 37 °C to facilitate spheroid formation [10]. Subsequently, the spheroids were transferred to PLL-coated glass-bottom dishes and incubated in DMEM with 10% FBS for an additional 24 h. Following washing with PBS(–), the spheroids were treated with DiD-labeled SAPSp-lipo, iRGD-lipo, or SAPSp-iRGD-lipo in a medium at pH 7.4 for 24 h and then fixed with 4% paraformaldehyde. Samples were mounted using VECTASHIELD^®^ with DAPI (Vector Laboratories, Inc., Newark, CA, USA) and examined using a confocal laser scanning microscope (CLSM; Nikon Corporation, Tokyo, Japan). Fluorescence intensity profiles were generated across the x–y planes at a depth of 4 μm using the NIS-Elements software (version 5.42.01; Nikon Corporation, Tokyo, Japan) [10]. To evaluate Neuropilin-1-mediated penetration, spheroids were pretreated with 30 μM EG3287 prior to treatment with DiO-labeled liposomes.

### 2.4. Immunofluorescent Staining of Neuropilin-1 in Tumors

B16-F1 cells (3.5 × 10^6^ cells) were combined with Matrigel at a 5:1 ratio and subcutaneously injected into the dorsal skin of 5-week-old hairless mice, as previously described [19,20]. Eight days after inoculation, when the mean tumor volume reached 280 mm^3^, DiO-labeled liposomes (50 µL) were administered into the tumor. After a 5 h interval, the tumors were excised, embedded in an OCT compound, and cryosectioned at a thickness of 16 µm using a LEICA CM 1100 (Leica Biosystems, Nussloch, Germany). The sections were fixed with 4% paraformaldehyde, permeabilized with PBS containing 1% Triton X-100, and blocked with Protein Block ((Dako, Agilent Technologies, Santa Clara, CA, USA). Subsequently, the sections were incubated overnight at 4 °C with an anti-Neuropilin-1 antibody, followed by staining with an Alexa Fluor^®^ 594 secondary antibody (Invitrogen™, Thermo Fisher Scientific Inc., Waltham, MA, USA) at 4 °C for 1 h. The sections were mounted using DAPI-containing VECTASHIELD^®^ and visualized using CLSM.

### 2.5. Actin Staining in Cells and Tumor Tissue

A375 cells were seeded onto poly-L-lysine (PLL)-coated chamber slides at a density of 3 × 10^4^ cells per well. Following a wash with PBS(–), the cells were exposed to SAPSp-lipo, iRGD-lipo, or SAPSp-iRGD-lipo in a pH 7.4 medium for one hour. For in vivo analysis, DiO-labeled liposomes were administered into the tumor tissue (50 µL), and the tumors were excised five hours post-injection, embedded in OCT, and cryosectioned to a thickness of 16 µm. Both cellular and tissue samples were fixed with 4% paraformaldehyde, permeabilized with 1% Triton X-100, and stained with rhodamine phalloidin according to the manufacturer’s instructions. Samples were mounted with DAPI-containing VECTASHIELD^®^ and analyzed using CLSM to observe actin polymerization.

### 2.6. ZO-1 Immunostaining

Caco-2 cells were seeded at a density of 2 × 10^5^ cells/well onto 0.002% poly-L-lysine (PLL)-coated 8-well chamber slides and incubated at 37 °C for 5 days, with medium changes every other day using Caco-2 Monolayer Formation Medium (Oriental Yeast Co., Ltd., Tokyo, Japan). After washing with PBS(−), the cells were treated with SAPSp-iRGD-lipo and incubated at 37 °C for 3 h. Following treatment, the cells were fixed with cold methanol, permeabilized with 1% Triton X-100, and blocked with 5% fetal bovine serum (FBS) in 0.1% Triton X-100/PBS(−) for 1 h at room temperature. After washing, the cells were incubated overnight at room temperature with a primary antibody solution containing rabbit polyclonal anti-ZO-1 IgG (4 µg/mL). Cells were then washed three times with 0.1% Triton X-100/PBS(−) and incubated with the secondary antibody, Alexa Fluor^®^ 488 goat anti-rabbit IgG (2 µg/mL), for 1 h at 37 °C in the dark. Finally, the samples were washed three times with 0.1% Triton X-100/PBS(−) and three times with ultrapure water, mounted with Vectashield^®^ Mounting Medium with DAPI (Vector Laboratories, Burlingame, CA, USA), and observed using CLSM.

### 2.7. Measurement of Transepithelial Electrical Resistance (TEER) in Caco-2 Cell Monolayers

Caco-2 cells were seeded onto 24-well cell culture inserts at a density of 6 × 10^4^ cells/insert and cultured with Caco-2 Monolayer Formation Medium (Oriental Yeast Co., Ltd., Tokyo, Japan). The culture medium was replaced every other day until the transepithelial electrical resistance (TEER) exceeded 500 Ω/cm^2^, indicating the formation of a tight monolayer. Subsequently, SAPSp-iRGD-lipo was added to the apical side of the monolayers, and TEER values were measured at 0, 10, 30, 60, and 180 min using a Millicell^®^ ERS-2 Volt-Ohm Meter (Merck Millipore, Darmstadt, Germany). As a positive control, EDTA treatment was used to induce tight junction disruption.

### 2.8. Biodistribution of Intravenously Administered Liposomes in Tumor-Bearing Mice

A suspension containing A375 cells (2 × 10^6^ cells/mouse) mixed 1:1 with Matrigel^®^ Basement Membrane Matrix (Corning Incorporated, Corning, NY, USA) was subcutaneously injected into the flank of BALB/cSlc-nu/nu mice. Eleven days after implantation, when the tumor volume had reached approximately 100 mm^3^, DiR-labeled liposomes (1 mol% DiR; total lipid dose, 1 µmol/mouse) were administered via the tail vein. In vivo biodistribution at 1 h and 24 h post-injection was assessed using the IVIS Lumina LT In Vivo Imaging System (PerkinElmer, Inc./Revvity Inc., Boston, MA, USA). Fluorescence intensities of the whole body and tumor regions were quantified using the system software. In addition, plasma samples collected at 1 h and 24 h after injection were diluted at least 50-fold with ethanol, and the fluorescence intensity of DiR was quantified using a microplate reader (Infinite M Plex, Tecan Group Ltd., Männedorf, Switzerland).

### 2.9. Cell Association and Intracellular Trafficking of Liposomes

B16-F1 cells (5 × 10^4^ cells/well) were incubated with rhodamine-labeled liposomes in serum-free medium at varying pH levels. Following a 1 h incubation at 4 °C, surface-bound liposomes were evaluated using flow cytometry. For trafficking analysis, cells were cultured in poly-L-lysine (PLL)-coated glass-bottom dishes and incubated with rhodamine-labeled liposomes at 37 °C. After 1 h, the endosomes/lysosomes and nuclei were stained with LysoTracker Green DND-26 and Hoechst 33342 (Invitrogen™, Thermo Fisher Scientific Inc., Waltham, MA, USA), respectively, and intracellular localization was analyzed using CLSM [8].

### 2.10. Evaluation of Cell Death

A375 cells (2.5 × 10^3^ cells/well) were seeded into poly-L-lysine (PLL)-coated 8-well chamber slides and incubated for 24 h. Following a wash with PBS(–), DMEM(–) was adjusted to pH 7.4, 6.5, or 6.0. SAPSp-iRGD-LNPs encapsulating KIF11 siRNA (final concentration 100 nM; 30 pmol/well) were used, with Lipofectamine 2000 serving as a positive control. After a 4 h incubation period, the medium was replaced with complete DMEM(+), and the cells were incubated for an additional 92 h. The cells were then fixed with 4% paraformaldehyde, washed, and mounted using VECTASHIELD^®^ with DAPI. Cell death was evaluated using CLSM.

### 2.11. Statistical Analysis

Statistical analyses were conducted using one-way ANOVA, followed by Tukey–Kramer HSD post hoc tests. Statistical significance was set at *p* < 0.05.

## 3. Results and Discussion

### 3.1. Comparison of Spheroid Penetration Between SAPSp- and SAPSp-iRGD-lipo

As shown in Table 1, the particle size of SAPSp-iRGD-lipo was approximately 100 nm. The surface charge of SAPSp-iRGD-lipo was measured as -15 mV at pH 7.4, which changed to −5.5 mV at pH 6.5. Similarly to SAPSp-lipo, the surface charge of SAPSp-iRGD-lipo transitioned from negative to near neutral under mildly acidic conditions. These findings indicate that the conjugation of the iRGD peptide sequence to SAPSp via a glycine linker does not compromise the pH-responsive charge-reversal capability of SAPSp [8].

Given that the internal microenvironment of spheroids can vary depending on the cell line employed, we assessed the internal environment of spheroids composed of either A375 or B16-F1 cells using a Hypoxia Probe. This probe demonstrated oxygen-dependent fluorescence quenching, with the fluorescence intensity being indicative of the oxygen concentration [21]. As depicted in Supplementary Figure S1, spheroids composed of A375 cells were more compact than those composed of B16-F1 cells and exhibited a stronger fluorescence of the Hypoxia Probe in their cores than in their peripheries. In addition, although detailed quantitative data on the ECM density of A375 tumors have not been reported, our spheroid model revealed that A375 spheroids exhibited a larger hypoxic core compared with B16-F1 spheroids (Figure S1). Since hypoxia is known to promote ECM remodeling and stiffening through the upregulation of collagen crosslinking enzymes such as lysyl oxidase and prolyl hydroxylase [22,23], this finding suggests that A375 tumors may possess a denser ECM microenvironment than B16-F1 tumors. Consistently, previous histological studies have reported a prominent collagen I and fibronectin deposition in A375-derived melanoma tissues [24,25], supporting the notion that A375 tumors exhibit a highly fibrotic and mechanically stiff ECM.

**Figure 1:**
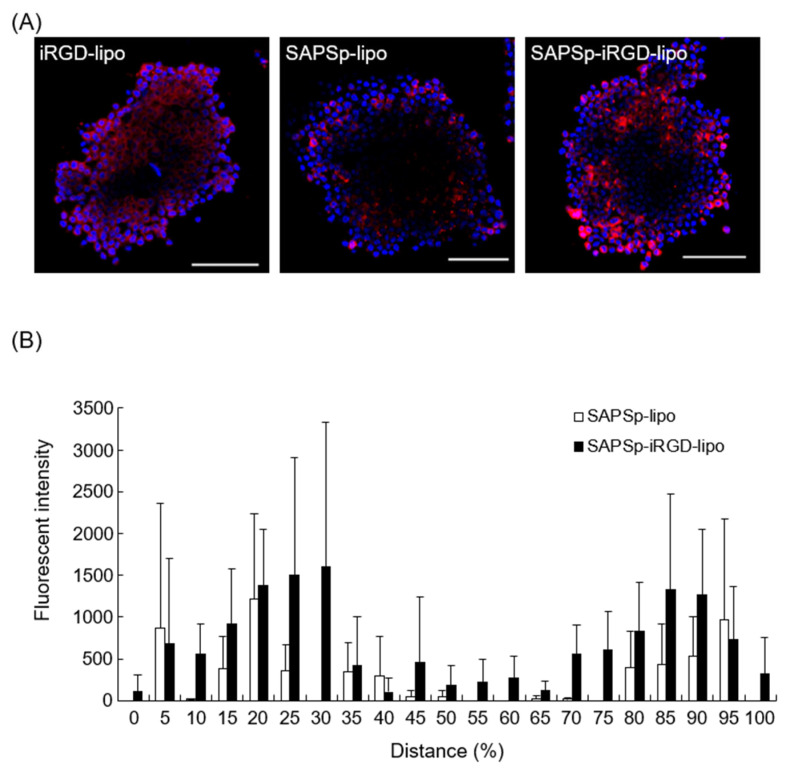
Spheroid penetration of iRGD-lipo and SAPSp-lipo. (**A**) Spheroids composed of A375 cells were treated with DiD-labeled iRGD-lipo, SAPSp-lipo, or SAPSp-iRGD-lipo and subsequently observed after 24 h using CLSM. Blue: nuclei (DAPI); red: liposomes (DiD). Scale bar: 100 µm. (**B**) A quantitative comparison of the spheroid penetration capability at pH 7.4 between SAPSp-lipo and SAPSp-iRGD-lipo was conducted using CLSM images of each sample in the x-y plane of three spheroids. The fluorescence distribution (%) of SAPSp-lipo and the total fluorescence intensity of SAPS-iRGD-lipo (white column) and SAPSp-iRGD-lipo (black column) in the x-y plane were determined at specified constant intervals (%) against the total fluorescence intensity on a line from a certain marginal region to the contralateral marginal region using a plot profile analysis. Data are presented as the mean ± SD from three spheroids. Distance (%) represents the relative position within the spheroid, where 50% corresponds to the spheroid core, and values smaller or larger than 50% indicate regions closer to the periphery.

Based on these observations, we used A375 cell-derived spheroids, which formed more compact structures, to evaluate the spheroid penetration of iRGD-lipo and SAPSp-lipo. In spheroids treated with iRGD-lipo, red fluorescence signals from the liposomes were observed even in the central region, suggesting that iRGD modification enhanced spheroid penetration (Figure 1A). Conversely, in spheroids treated with SAPSp-lipo, the liposomes remained localized near the peripheral region (Figure 1A). However, the spheroids treated with SAPSp-iRGD-lipo also exhibited red fluorescence signals in the central regions, similar to those observed following iRGD-lipo treatment (Figure 1A).

The semi-quantitative analysis based on CLSM images revealed that SAPSp-iRGD-lipo was more widely distributed in the core of the spheroid than SAPSp-lipo (Figure 1B). Even when evaluated using B16-F1 cell-derived spheroids, which form looser structures, SAPSp-iRGD-lipo penetrated deeper than SAPSp-lipo (Supplementary Figure S2). Collectively, these findings suggest that SAPSp-iRGD-lipo demonstrates superior spheroid penetration compared to SAPSp-lipo.

### 3.2. Involvement of Neuropilin-1 in the Penetration of SAPSp-iRGD-lipo

**Figure 2:**
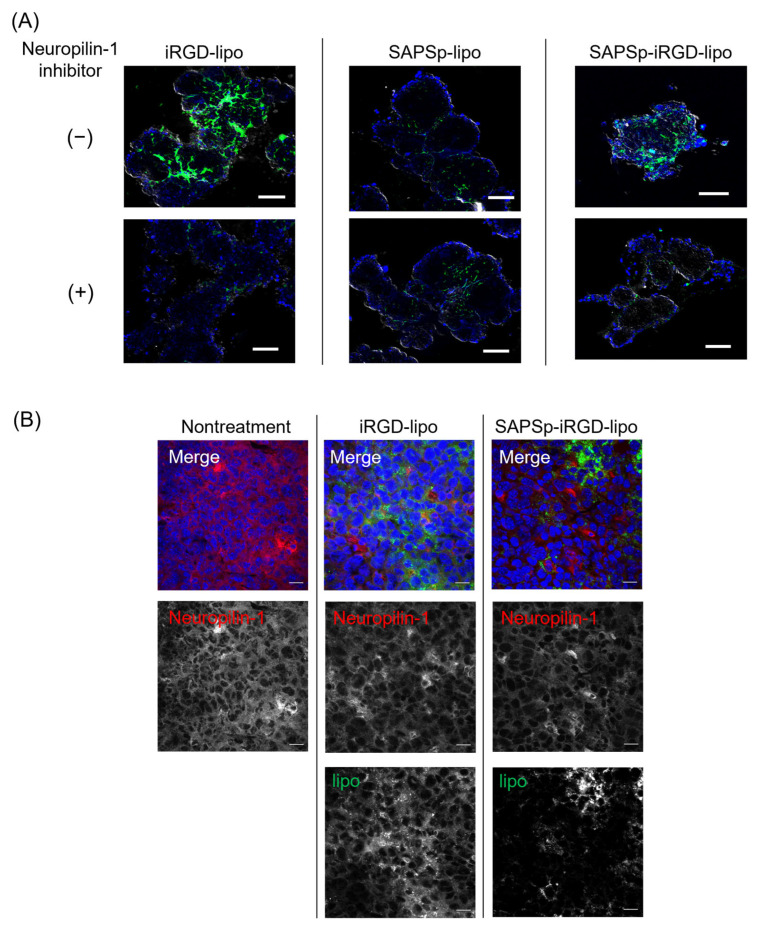
Relationship between the tissue penetration of SAPSp-iRGD-lipo and Neuropilin-1. (**A**) Effect of Neuropilin-1 inhibitor on the spheroid penetration of SAPSp-iRGD-lipo. Spheroids composed of A375 cells were pre-incubated with the Neuropilin-1 inhibitor EG3287 (final concentration: 30 µM) for 30 min. Subsequently, DiO-labeled iRGD-lipo, SAPSp-lipo, or SAPSp-iRGD-lipo was added, and spheroids were observed after 24 h using CLSM. Blue: nuclei (DAPI); green: liposomes (DiO). Scale bar: 100 µm. (**B**) Intratumoral distribution of Neuropilin-1 expression and liposomes. DiO-labeled iRGD-lipo or SAPSp-iRGD-lipo was locally administered into tumors. After 5 h, the tumors were excised, fixed with 4% formaldehyde, and subjected to immunostaining, followed by CLSM. Blue: nuclei (DAPI); green: liposomes (DiO); red: Neuropilin-1 (Alexa Fluor 594). Scale bar: 20 µm.

The iRGD peptide facilitates tissue penetration by activating Neuropilin-1 [26]. To determine whether the enhanced penetration of SAPSp-iRGD-lipo was due to iRGD conjugation, we assessed spheroid penetration in the presence of a Neuropilin-1 inhibitor. A co-treatment with the NRP-1 inhibitor EG3287 markedly suppressed the penetration of both iRGD-lipo and SAPSp-iRGD-lipo into the spheroids (Figure 2A and Supplementary Figure S3). In contrast, the penetration of SAPSp-lipo was unaffected by the inhibitor. These findings indicate that the enhanced spheroid penetration of SAPSp-iRGD-lipo is mediated, at least in part, by an NRP-1-dependent mechanism. To further validate the involvement of NRP-1 in tissue penetration in vivo, we examined the correlation between the liposome distribution and NRP-1 expression following intratumoral injection in tumor-bearing mice. As shown in Figure 2B, both iRGD-lipo and SAPSp-iRGD-lipo exhibited green fluorescence signals that were predominantly localized in regions with a high NRP-1 expression. A quantitative co-localization analysis revealed Pearson’s correlation coefficients of 0.464 and 0.442, respectively, indicating a moderate yet specific molecular interaction between the iRGD motif and NRP-1-expressing tumor regions. Collectively, these results demonstrate that the enhanced penetration of SAPSp-iRGD-lipo arises from its NRP-1-mediated, pH-responsive dual functionality, integrating active tumor targeting via iRGD with the pH-dependent charge conversion properties conferred by the SAPSp segment. This dual mechanism likely contributes to the improved intratumoral diffusion observed in both spheroid and tumor models.

### 3.3. Involvement of Actin Depolymerization in the Tissue Penetration of SAPSp-iRGD-lipo

In our previous study, we demonstrated that SAPSp-lipo facilitates tissue penetration by inducing actin depolymerization [10]. To ascertain whether actin depolymerization contributed to the enhanced tissue penetration of SAPSp-iRGD-lipo, we investigated actin dynamics in a monolayer culture system.

**Figure 3:**
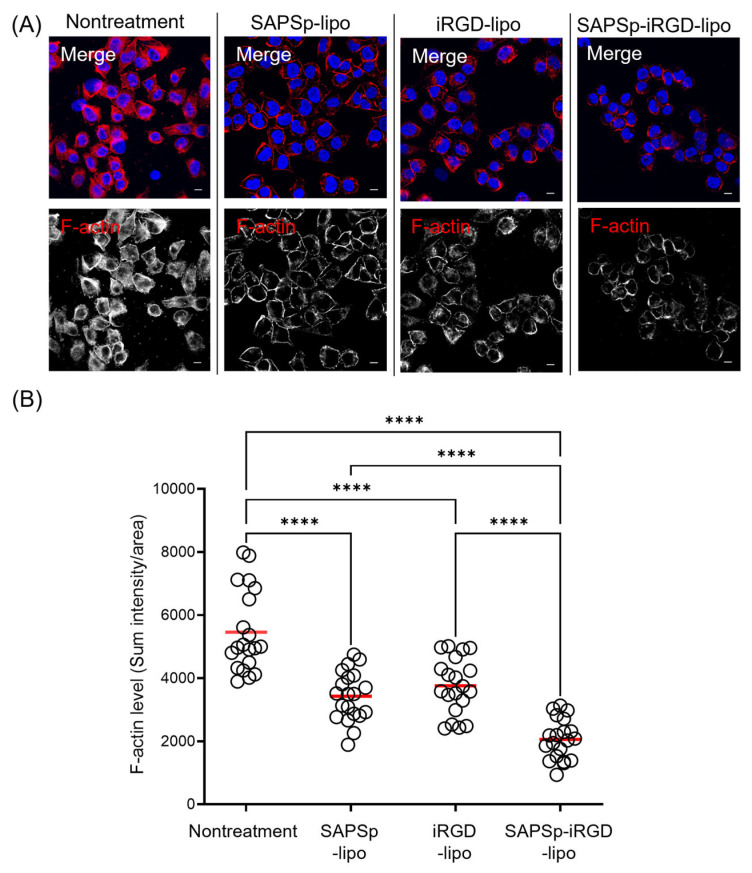
Actin depolymerization induced by SAPSp-iRGD in monolayered cells. A375 cells were exposed to liposomes for 1 h, followed by F-actin staining and observation using CLSM. (**A**) Representative fluorescence images. Blue: nuclei (DAPI); red: F-actin (rhodamine-phalloidin). Scale bar = 10 µm. (**B**) Semi-quantitative analysis of F-actin fluorescence intensity per cell. Each circle represents the fluorescence intensity of an individual cell, and the red bar indicates the mean value. *n* = 20, **** *p* < 0.0001.

Actin depolymerization was indicated by a reduction in the red fluorescence of F-actin staining. Consistent with prior findings [10], treatment with SAPSp-lipo resulted in a significant reduction in F-actin fluorescence compared to that in untreated cells, confirming its actin depolymerizing activity. Similarly, SAPSp-iRGD-lipo treatment led to a notable decrease in F-actin staining, suggesting that SAPSp-iRGD-lipo retained the actin depolymerizing activity of SAPSp-lipo. Furthermore, we quantified the fluorescence intensity of F-actin and found that SAPSp-iRGD-lipo treatment induced a stronger reduction in F-actin compared to SAPSp-lipo or iRGD-lipo alone (Figure 3B). The calculated Bliss Excess was 5.6%, indicating a mild synergistic effect between the SAPSp and iRGD motifs (Table 2). To distinguish whether this synergistic effect originated from the covalent (chimeric) linkage of SAPSp and iRGD or from a simple physical combination, we prepared additional liposomes co-modified with SAPSp (5 mol%) and iRGD (5 mol%) without covalent linkage. Their physicochemical properties are summarized in Supplementary Table S1. The ζ-potentials of these co-modified liposomes remained nearly neutral at both pH 7.4 and pH 6.5, contrasting with the pH-dependent charge reversal observed in SAPSp-iRGD-lipo, which shifts from negative (pH 7.4) to positive (pH 6.5). These results indicate that the chimeric linkage between SAPSp and iRGD is essential for integrating pH responsiveness and tumor-penetrating ability within a single molecular design.

**Figure 4:**
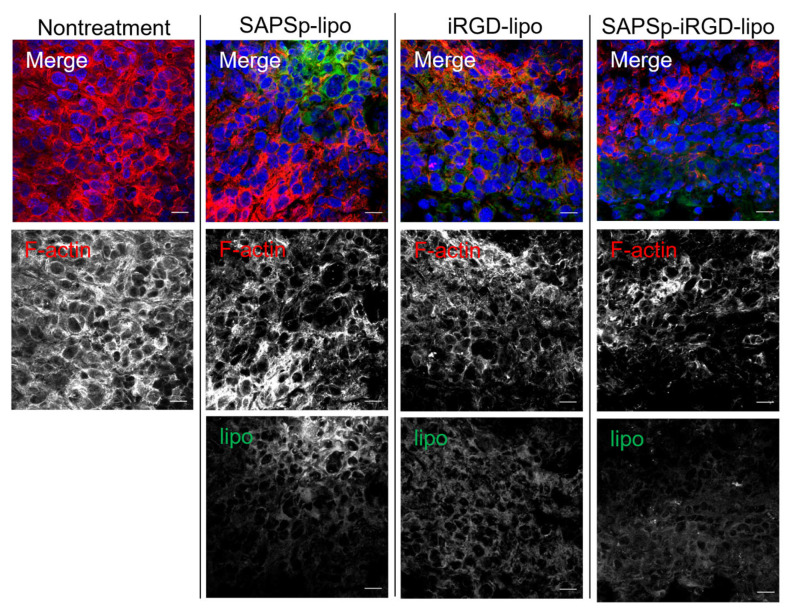
Relationship between intratumoral localization of liposomes and F-actin expression. SAPSp-lipo, iRGD-lipo, and SAPSp-iRGD-lipo were labeled with DiO and locally administered to tumor tissues. After 5 h, tumors were excised, fixed with 4% formaldehyde, and stained with rhodamine-labeled phalloidin for observation using CLSM. Blue: nuclei (DAPI); green: liposomes (DiO); red: F-actin (rhodamine-phalloidin). Scale bar = 20 µm.

To evaluate the role of actin depolymerization in the in vivo tissue penetration of SAPSp-iRGD-lipo, we analyzed its distribution in tumor-bearing mice following intratumoral injection. Similarly to SAPSp-lipo, SAPSp-iRGD-lipo exhibited a preferential diffusion into tumor regions characterized by a low F-actin expression (Figure 4). To further investigate the spatial relationship between liposomal localization and the actin cytoskeleton, we analyzed the co-localization between F-actin and each liposome formulation. The Pearson’s correlation coefficients were 0.218 ± 0.021 for SAPSp-lipo, 0.247 ± 0.028 for iRGD-lipo, and 0.257 ± 0.029 for SAPSp-iRGD-lipo, confirming the reduced spatial overlap between F-actin and SAPSp-iRGD-lipo (Table 3). These results support the notion that actin depolymerization contributes to the enhanced tissue penetration of SAPSp-iRGD-lipo. In conjunction with the results presented in Figure 2, Figure 3 and Figure 4, these findings indicate that SAPSp-iRGD-lipo achieves substantial tissue penetration through the synergistic mechanisms of SAPSp-mediated actin depolymerization and iRGD-mediated, Neuropilin-1-dependent transcytosis [15,16].

### 3.4. Effect of SAPSp-iRGD-lipo on the Intercellular Barrier

**Figure 5:**
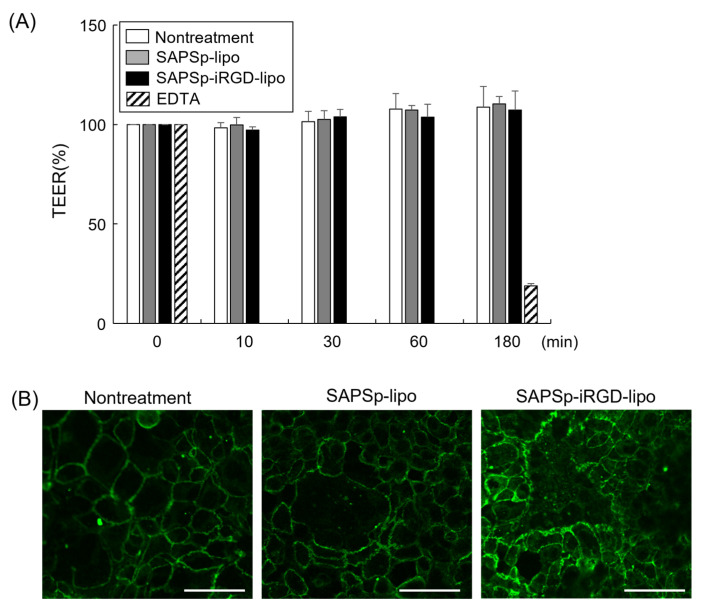
Effect of SAPSp-iRGD-lipo on intercellular barrier. (**A**) Caco-2 cell monolayers were treated with either SAPSp-lipo or SAPSp-iRGD-lipo, and alterations in transepithelial electrical resistance (TEER) were assessed. Data are expressed as percentages relative to the TEER value at 0 min. Data are presented as mean ± SD (n = 3). Treatment with 50 mM EDTA was used as a positive control. (**B**) Caco-2 cell monolayers were treated with either SAPSp-lipo or SAPSp-iRGD-lipo for 3 h. Following immunostaining with an anti-ZO-1 antibody, samples were examined using CLSM. Green: ZO-1, Scale bar: 20 µm.

The penetration of nanoparticles into tissues typically occurs via two principal routes: the paracellular pathway, which involves a movement through intercellular gaps, and transcytosis, which entails a passage through the cells [27,28]. Owing to the technical challenges associated with the direct and quantitative assessment of the contribution of transcytosis, our investigation focused on evaluating the role of the paracellular pathway in the penetration of SAPSp-iRGD-lipo. For nanoparticles to navigate tissues via the paracellular route, intercellular junctions must open. To determine whether SAPSp-iRGD-lipo induced the opening of intercellular junctions, we measured the transepithelial electrical resistance (TEER) in Caco-2 monolayers [29]. The results indicated no significant changes in TEER following treatment with either SAPSp-lipo or SAPSp-iRGD-lipo (Figure 5A), suggesting that neither formulation compromised the epithelial barrier integrity.

Furthermore, the disruption of tight junctions correlates with an altered localization of the tight junction protein Zo-1 [30,31]. Consequently, we performed immunostaining using an anti-Zo-1 antibody to assess the tight junction morphology. The findings revealed that Zo-1 localization remained consistent after treatment with either SAPSp-lipo or SAPSp-iRGD-lipo compared to the untreated controls. These results imply that the paracellular pathway does not constitute a major route for the tissue penetration of SAPSp-iRGD-lipo nanoparticles.

### 3.5. Comparison of Biodistribution of Intravenously Administered Liposomes in Tumor-Bearing Mice

**Figure 6:**
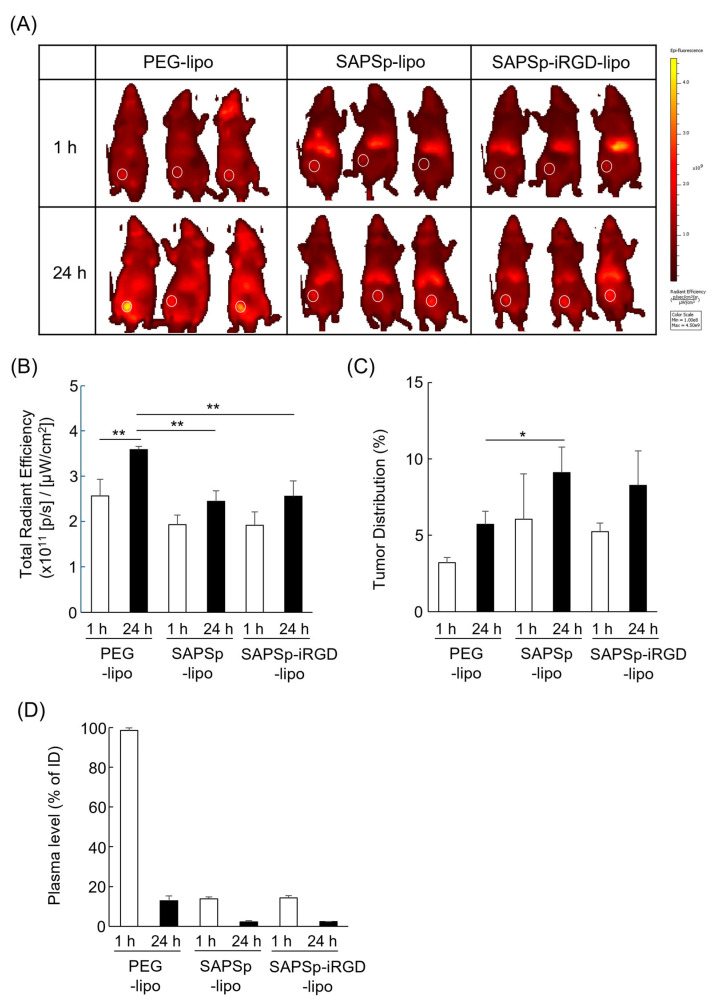
Biodistribution of intravenously administered liposomes in tumor-bearing mice. A375 tumor-bearing mice were intravenously injected with DiR-labeled liposomes. In vivo fluorescence images were acquired at 1 h and 24 h post-injection using an IVIS Lumina LT imaging system. (**A**) Representative fluorescence images. White circles indicate tumor regions. (**B**,**C**) Quantification of fluorescence intensities in whole body and tumor regions, respectively, using the system software. (**D**) Plasma fluorescence at 1 h and 24 h was measured using a microplate reader. n = 3, * *p* < 0.05, ** *p* < 0.01.

To examine whether SAPSp-iRGD-lipo retains its functional properties and systemic stability in vivo, DiR-labeled liposomes were intravenously administered to tumor-bearing mice, and their biodistribution was analyzed using an IVIS imaging system. As shown in Figure 6A–C, SAPSp-iRGD-lipo exhibited a biodistribution pattern similar to that of SAPSp-lipo. The total fluorescence intensity in mice treated with SAPSp-iRGD-lipo or SAPSp-lipo was lower than that of PEG-lipo, which served as a long-circulating control, and both formulations accumulated predominantly in the liver. A quantitative analysis of plasma samples (Figure 6D) revealed that the blood concentrations of SAPSp-iRGD-lipo and SAPSp-lipo were approximately 20% of the injected dose at 1 h post-injection. Previous pharmacokinetic studies have shown that anionic liposomes typically retain approximately 10–30% of the injected dose in the bloodstream at 1 h post-injection, whereas cationic liposomes exhibit much lower blood levels, often below 10% of the injected dose [32,33,34]. Consistent with these reports, the blood retention of SAPSp-iRGD-lipo and SAPSp-lipo falls within the range expected for anionic liposomes, suggesting that the surface-modified peptides remain stably associated with the liposomal surface under in vivo conditions. Importantly, both SAPSp-iRGD-lipo and SAPSp-lipo accumulated in tumor tissues as early as 1 h post-injection, whereas PEG-lipo showed a gradual tumor accumulation at 24 h. Taken together, these results demonstrate that SAPSp-iRGD-lipo maintains its in vivo stability and achieves a rapid tumor accumulation through active targeting driven by pH-responsive surface charge conversion, rather than passive accumulation via the enhanced permeability and retention (EPR) effect.

### 3.6. Cytosolic Delivery of Cargo by SAPSp-iRGD-Modified Nanoparticles

**Figure 7:**
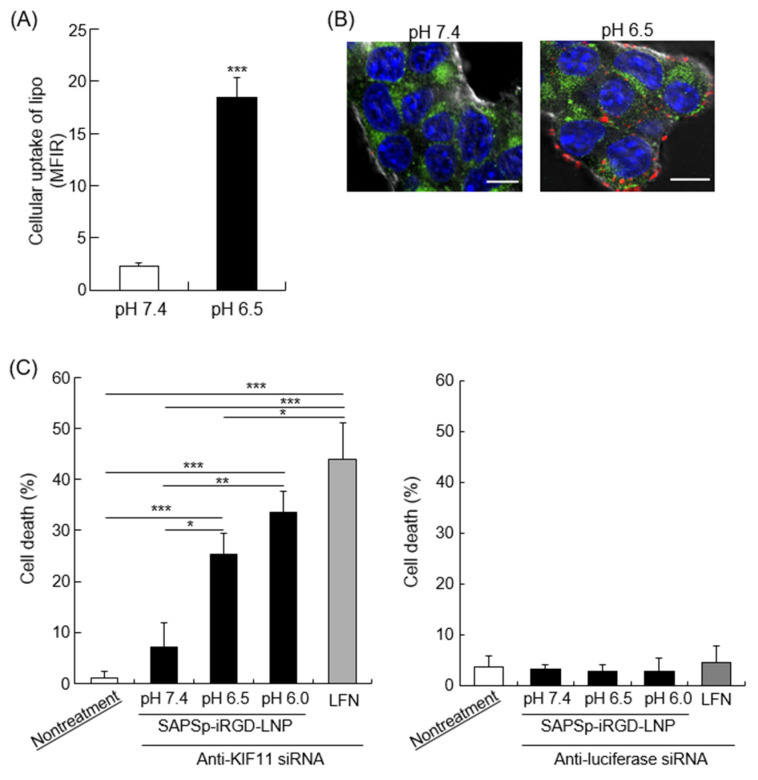
Intracellular trafficking and cargo delivery of SAPSp-iRGD-lipo. (**A**) Cells were exposed to rhodamine-labeled SAPSp-iRGD-lipo at pH 7.4 or 6.5 for 1 h at 4 °C, and the binding of liposomes to the cells was assessed via flow cytometry. (**B**) Cells were treated with rhodamine-labeled SAPSp-iRGD-lipo at pH 7.4 or 6.5 for 1 h at 37 °C, and the intracellular distribution of the liposomes was examined using CLSM. Blue: nuclei (Hoechst 33342); green: endosomes/lysosomes (LysoTracker Green DND-26); red: SAPSp-iRGD. Scale bar: 10 µm. (**C**) Induction of cell death through anti-KIF11 siRNA delivery by SAPSp-iRGD-LNPs. Cells were treated with SAPSp-iRGD-LNPs encapsulating anti-KIF11 siRNA at pH 7.4, 6.5, and 6.0 for 96 h. After fixation with 4% paraformaldehyde, the nuclei were stained with DAPI and observed using CLSM. Apoptotic cells in the CLSM images were quantified, and their percentages relative to the total number of cells were calculated. Anti-luciferase siRNA was used as a control siRNA. Data are presented as mean ± SD. (* *p* < 0.05; ** *p* < 0.01; *** *p* < 0.001; n = 3).

Our previous research demonstrated that SAPSp-modified liposomes respond to a mildly acidic tumor microenvironment, facilitating cargo delivery into the cytosol [8]. To ascertain whether SAPSp-iRGD-lipo exhibited a pH-responsive cytosolic delivery capability akin to that of SAPSp-lipo, we examined its cellular uptake and intracellular localization. As illustrated in Figure 7A, SAPSp-iRGD-lipo displayed a significantly enhanced cellular uptake at pH 6.5 compared to that at pH 7.4, with the magnitude of this increase comparable to that observed with SAPSp-lipo [8]. Furthermore, confocal laser scanning microscopy (CLSM) revealed a minimal red fluorescence (indicative of liposome presence) within cells at pH 7.4, whereas a pronounced intracellular red fluorescence was observed at pH 6.5, indicating an enhanced uptake under mildly acidic conditions. Importantly, the red fluorescent signal from SAPSp-iRGD-lipo did not colocalize with endosomal or lysosomal markers at pH 6.5, suggesting a successful delivery to the cytosol rather than a retention in endocytic compartments [8]. These findings suggest that SAPSp-iRGD-lipo, similar to SAPSp-lipo, possesses a pH-responsive cellular uptake and cytosolic delivery capabilities in mildly acidic conditions.

To further verify whether SAPSp-iRGD modification confers a cytosolic delivery ability, we prepared lipid nanoparticles (LNPs) encapsulating apoptosis-inducing siRNA and modified their surfaces with SAPSp-iRGD (SAPSp-iRGD-LNPs). Kinesin family member 11 (KIF11), a motor protein involved in mitosis, plays a significant role in cancer cells by influencing tumor progression, prognosis, and therapeutic responses [35]. The inhibition of KIF-11 activates the intrinsic apoptotic pathway [36]. In this study, anti-KIF11 siRNA was encapsulated in LNPs. As presented in Table 4, SAPSp-iRGD-LNPs encapsulating either anti-KIF11 or control siRNA exhibited a surface charge shift from negative to positive as the pH decreased from 7.4 to 6.5, similar to that of SAPSp-iRGD-lipo. Although the encapsulation efficiencies of anti-KIF11 siRNA (85.0%) and control siRNA (72.7%) differed slightly, both formulations showed comparable particle sizes and ζ-potentials, indicating that the physicochemical properties and pH-responsive charge-reversal behavior of SAPSp-iRGD-LNPs were unaffected by the type of encapsulated siRNA. To eliminate any influence of encapsulation efficiency on the cytotoxicity results, the siRNA dose was standardized to 30 pmol per well for both formulations. Therefore, the difference in cytotoxicity observed in Figure 7C can be attributed to the gene-specific knockdown of KIF11 rather than to variations in the encapsulation efficiency. Moreover, as depicted in Supplementary Figure S4, fluorescently labeled siRNA-loaded SAPSp-iRGD-LNPs demonstrated a greater spheroid penetration than SAPSp-LNPs, indicating that SAPSp-iRGD enhanced tissue penetration even when conjugated to structurally more rigid LNPs than liposomes. As shown in Figure 7C, under mildly acidic conditions (pH 6.5 and 6.0), the SAPSp-iRGD-LNP-mediated delivery of anti-KIF11 siRNA resulted in a significant induction of cell death, whereas the control siRNA delivery did not induce cell death under these conditions. To further compare the siRNA delivery performance between SAPSp-LNP and SAPSp-iRGD-LNP, fluorescently labeled siRNA-loaded nanoparticles were analyzed using CLSM. Both formulations exhibited a markedly enhanced cellular uptake under acidic conditions (pH ≤ 6.5; Supplementary Figure S5). Notably, the intracellular fluorescence of siRNA delivered by SAPSp-iRGD-LNP was slightly lower than that of SAPSp-LNP, suggesting a marginally reduced in vitro delivery efficiency and consequently a weaker gene-knockdown effect. However, considering that SAPSp-iRGD-LNP demonstrated a superior tissue penetration in spheroids (Supplementary Figure S4) and tumors, its enhanced intratumoral distribution is expected to compensate for this minor difference in cellular uptake, potentially yielding a higher overall antitumor gene-silencing effect in vivo. To further evaluate the therapeutic potential of SAPSp-iRGD-LNPs, we examined their biodistribution and tumor-delivery behavior following intravenous administration. As shown in Figure 6, both SAPSp-lipo and SAPSp-iRGD-lipo achieved an efficient tumor delivery, although their overall accumulation was lower than that of PEG-lipo. These results indicate that SAPSp-based systems exhibit active targeting properties mediated by their pH-responsive surface charge conversion, yet further optimization of their pharmacokinetic and tumor-delivery profiles will be necessary to translate their enhanced penetration into a measurable in vivo therapeutic efficacy. Given these findings, we consider the present results as a foundational step toward developing an optimized SAPSp-iRGD-LNP formulation capable of demonstrating improved antitumor outcomes in future studies. In comparison with previous reports, various nanoparticle systems have been developed to enhance intratumoral delivery, including pH-responsive charge-conversion systems [37,38], tumor-penetrating peptide-modified carriers [39,40], and integrin-targeting or ECM-degrading approaches [41,42]. These formulations typically rely on either (i) pH-triggered activation or (ii) receptor-mediated internalization to promote tumor penetration. In contrast, the present SAPSp-iRGD system integrates both mechanisms within a single chimeric peptide, combining the slightly acidic pH-responsive charge reversal of SAPSp with the Neuropilin-1-dependent tissue penetration of iRGD. This design differs from previously reported systems using mixed or co-administered peptides [43,44], where cooperative functionality can be limited by a heterogeneous surface presentation. In our study, SAPSp-iRGD-LNPs exhibited a coordinated pH-responsiveness and active tumor penetration without altering the particle size or peptide density, demonstrating that the covalent linkage of SAPSp and iRGD yields a synergistic functionality. Thus, SAPSp-iRGD-LNPs represent a distinct class of dual-functional, microenvironment-responsive nanocarriers that unify pH-triggered activation and receptor-mediated penetration, providing a mechanistic bridge to extend existing tumor penetration strategies.

## 4. Conclusions

To enhance the tissue penetration capability of the tumor pH-responsive peptide SAPSp, we developed an innovative peptide, SAPSp-iRGD, by conjugating the tissue-penetrating peptide sequence iRGD to the C-terminus of SAPSp. Nanoparticles modified with SAPSp-iRGD retained the pH-responsive properties of SAPSp and demonstrated a significantly improved tissue penetration. Moreover, our findings indicate that this enhanced penetration involves both neuropilin-1-mediated pathways and actin depolymerization. Owing to their combined tumor-specific tissue penetration and mildly acidic pH responsiveness, SAPSp-iRGD-modified nanoparticles represent a promising novel delivery system for targeted nucleic acid delivery into deep tumor regions. Although SAPSp-iRGD-modified nanoparticles demonstrated an enhanced tumor penetration and pH-responsive delivery, further studies are required to evaluate their pharmacokinetic profile and therapeutic efficacy in multiple tumor models. In addition, the optimization of peptide density and assessment of safety and large-scale production will be important for future clinical translation.

## Figures and Tables

**Table 1:** Physicochemical properties of SAPSp-iRGD liposomes.

pH	Particle Size (nm)	Polydispersity Index	ζ-potential (mV)
7.4	103 ± 12	0.262 ± 0.001	−15 ± 1.8
6.5	103 ± 11	0.341 ± 0.016	−5.5 ± 1.3

**Table 2:** Comparison between observed and Bliss-predicted actin depolymerization efficiencies following liposome treatments.

Treatment	Mean %	Bliss-Predicted	Bliss Excess	Interaction
**SAPSp-lipo**	37.2	-	-	-
**iRGD-lipo**	31.1	-	-	-
**SAPSp-iRD-lipo**	62.3	56.7	+5.6	Mild synergy

**Table 3:** Evaluation of the co-localization between intratumoral distribution of various liposomes and F-actin-stained regions.

	SAPSp-lipo	iRGD-lipo	SAPSp-iRGD-lipo
**Pearson’s correlation coefficient**	0.218 ± 0.021	0.247 ± 0.028	0.257 ± 0.029

**Table 4:** Physicochemical properties of SAPSp-iRGD-LNP encapsulating siRNAs.

siRNA Encapsulated in LNPs	Encapsulation (%)	pH	Particle Size (nm)	Polydispersity Index	ζ-Potential (mV)
Anti-KIF11 siRNA	85.0	7.4	73.4 ± 9.2	0.353 ± 0.080	−16 ± 2.5
		6.5	74.5 ± 11	0.353 ± 0.053	−6.7 ± 1.9
Control siRNA	72.7	7.4	70.0 ± 5.1	0.297 ± 0.061	−13 ± 3.1
		6.5	72.9 ± 21	0.312 ± 0.119	−6.4 ± 3.2

## Data Availability

The original contributions presented in this study are included in the article/Supplementary Materials. Further inquiries can be directed to the corresponding author.

## Supplementary Materials

The following supporting information can be downloaded at https://www.mdpi.com/article/10.3390/nano15221695/s1. Table S1: Physicochemical properties of liposomes co-modified with stearyl-SAPSp and stearyl-iRGD; Figure S1: Comparison of the hypoxic microenvironment within spheroids composed of A375 cells and those composed of B16-F1 cells; Figure S2: Quantitative comparison of the permeability of SAPSp-lipo and SAPSp-iRGD-lipo in B16-F1 cell-derived spheroids; Figure S3: Surface intensity plot showing the effect of Neuropilin-1 inhibition on the spheroid penetration of SAPSp-iRGD-lipo; Figure S4: Spheroid penetration of SAPSp-LNP and SAPSp-iRGD-LNP. Figure S5: Cellular uptake of SAPSp-LNP and SAPSp-iRGD-LNP.

## Abbreviations

The following abbreviations are used in this manuscript:

lipo	Liposomes
SAPSp	Slightly acidic pH-sensitive peptides
ECM	Extracellular matrix
iRGD	Internalizing RGD
LNPs	Lipid nanoparticles
PEG	Polyethylene glycol
EPR	Enhanced permeability and retention
KIF11	Kinesin family member 11
CLSM	Confocal laser scanning microscopy
TEER	Transepithelial electrical resistance
