# Supplementary material for: Development of Tumor Microenvironment-Responsive Nanoparticles with Enhanced Tissue Penetration
Source: Nanomaterials (Basel). 2025 Nov 9;15(22):1695. doi: 10.3390/nano15221695 (PMC12655548; doi:10.3390/nano15221695)
Supplement: Supplementary file 1 [file nanomaterials-15-01695-s001.zip › nanomaterials-3771296-supplementary.pdf]

Table S1. Physicochemical properties of liposomes co-modified with stearyl-SAPSp and stearyl-iRGD

pH	Particle size (nm)	Polydispersity index	ζ-potential (mV)
7.4	95.6 ± 16.4	0.413 ± 0.098	−0.54 ± 4.6
6.5	92.7 ± 10.3	0.370 ± 0.027	−1.8 ± 3.6

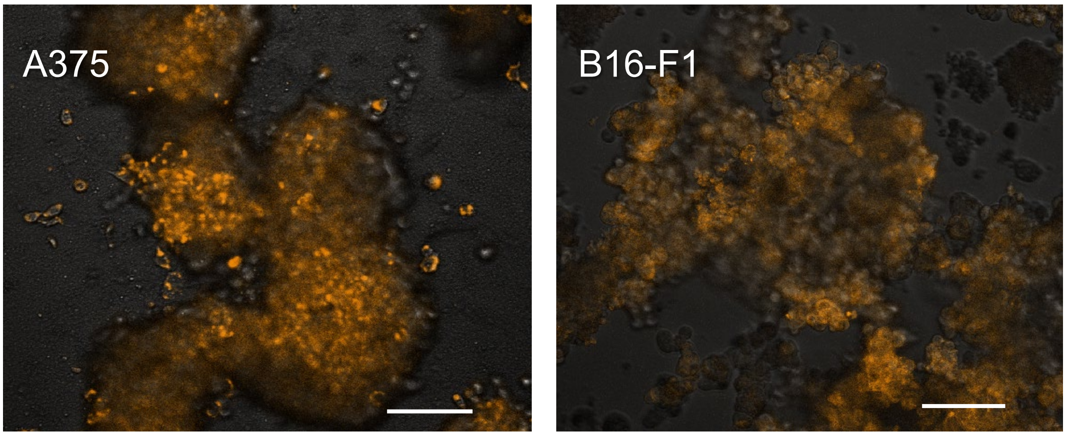

Figure S1 Comparison of the hypoxic microenvironment within spheroids composed of A375 cells and those composed of B16-F1 cells. Spheroids composed of A375 cells and those composed of B16-F1 cells were stained with a hypoxia probe, and hypoxic regions were visualized using confocal laser scanning microscopy (CLSM). Orange indicates hypoxic areas. Scale bar: 100 μm.

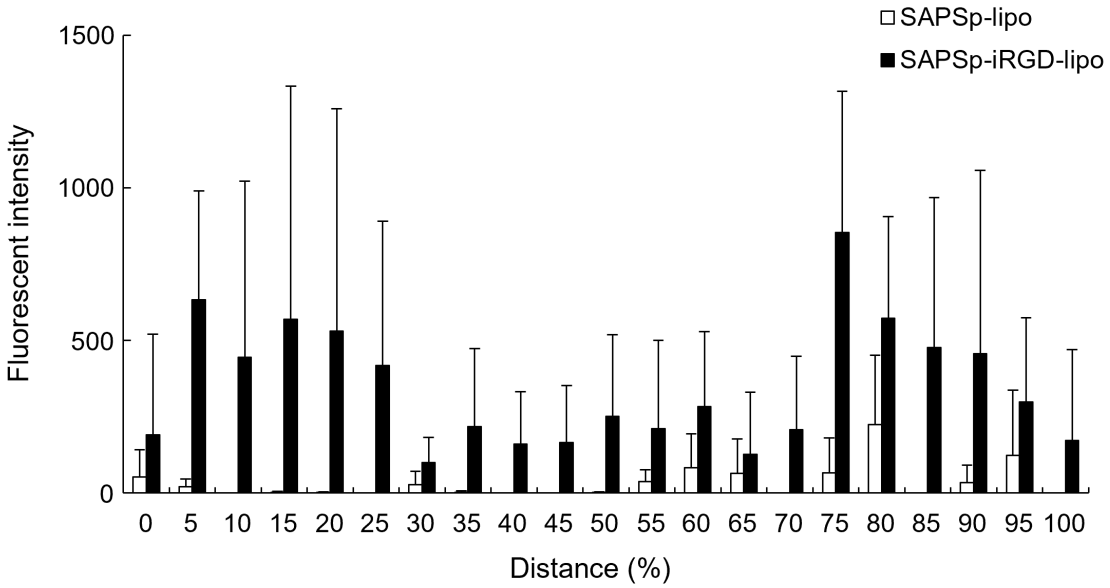

Figure S2 Quantitative comparison of the permeability of SAPSp-lipo and SAPSp-iRGD-lipo in B16-F1 cell-derived spheroids. A quantitative comparison of the spheroid penetration capability at pH 7.4 between SAPSp-lipo and SAPSp-iRGD-lipo was conducted using CLSM images of each sample in the x-y plane of three spheroids. The fluorescence distribution (%) of SAPSp-lipo and the total fluorescence intensity of SAPS-iRGD-lipo (white column) and SAPSp-iRGD-lipo (black column) in the x–y plane were determined at specified constant intervals (%) against the total fluorescence intensity on a line from a certain marginal region to the contralateral marginal region using a plot profile analysis. Data are presented as mean ± SD from three spheroids

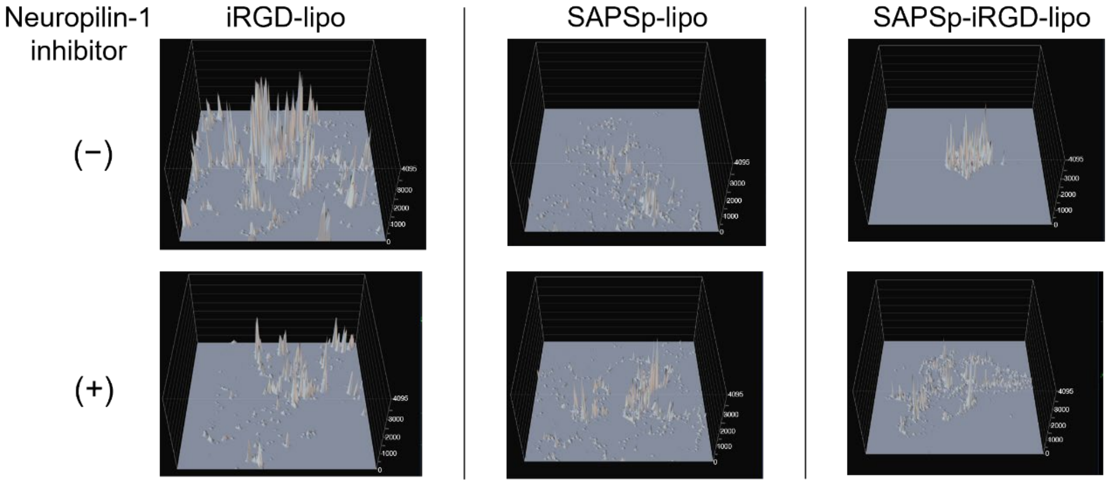

Figure S3 Surface intensity plot showing the effect of Neuropilin-1 inhibition on the spheroid penetration of SAPSp-iRGD-lipo. A375 cell spheroids were pre-incubated with the Neuropilin-1 inhibitor EG3287 (final concentration: 30 μM). Subsequently, DiO-labeled iRGD-lipo, SAPSp-lipo, or SAPSp-iRGD-lipo was added, and spheroids were observed after 24 h using confocal laser scanning microscopy (CLSM). Surface intensity profiles were generated using NIS-Elements AR analysis software.

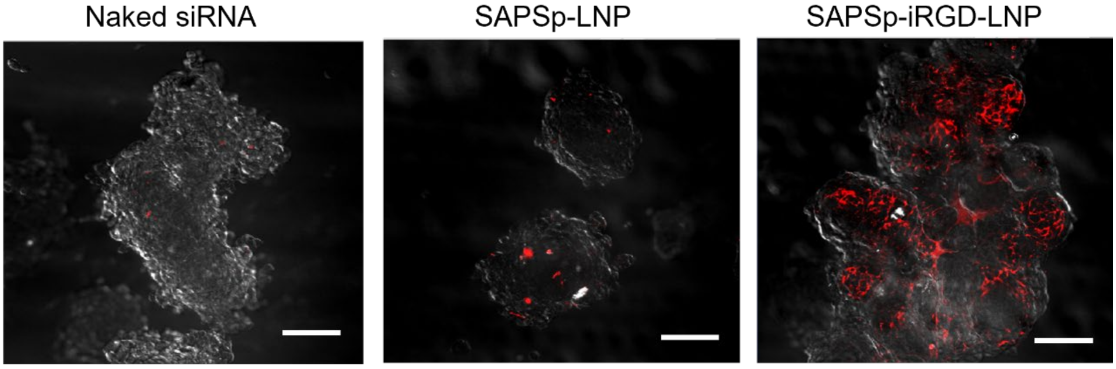

Figure S4 Spheroid Penetration of SAPSp-LNP and SAPSp-iRGD-LNP. Spheroids composed of A375 cells were treated with LNPs encapsulating Alexa 546-labeled siRNA and subsequently observed after 24 h using confocal laser scanning microscopy (CLSM). Red: siRNA (Alexa 546). Scale bar: 100 μm.

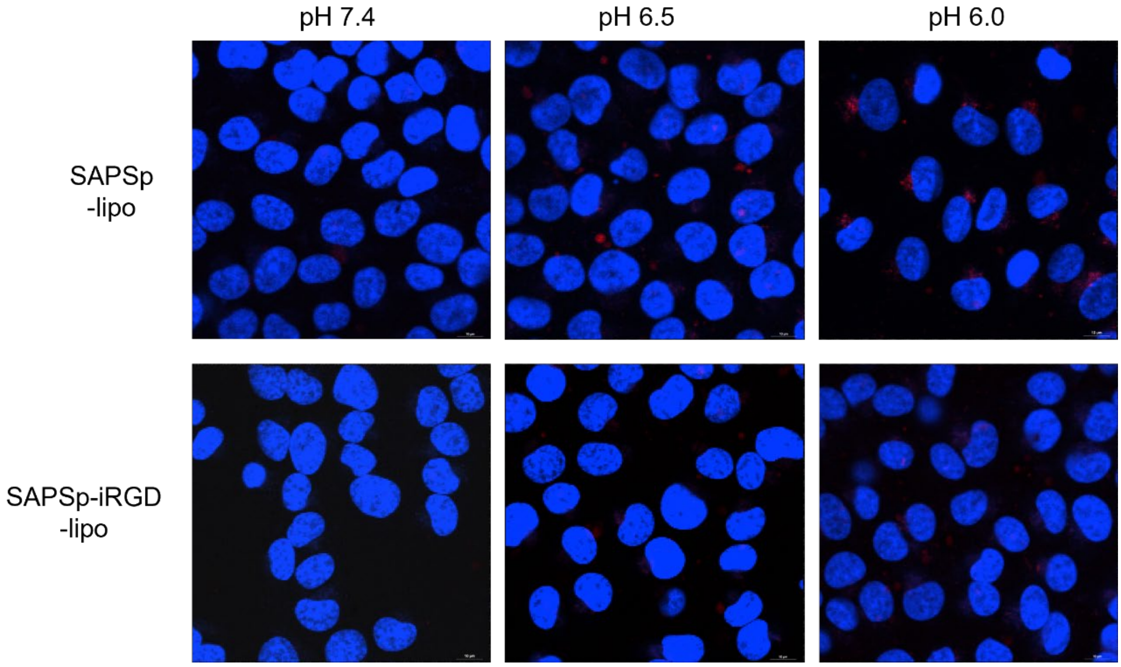

Figure S5 Cellular uptake of SAPSp-LNP and SAPSp-iRGD-LNP. A375 cells were treated with LNPs encapsulating Alexa 546-labeled siRNA and subsequently observed after 1 h using confocal laser scanning microscopy (CLSM). Blue: Hoechst33342, Red: siRNA (Alexa 546).
